# Cancer Immunotherapy: Current and Future Perspectives on a Therapeutic Revolution

**DOI:** 10.3390/jcm10225246

**Published:** 2021-11-11

**Authors:** Alessandro Rizzo, Veronica Mollica, Matteo Santoni, Francesco Massari

**Affiliations:** 1 Medical Oncology, IRCCS Azienda Ospedaliero-Universitaria di Bologna, Via Albertoni-15, 40121 Bologna, Italy; veronica.mollica7@gmail.com (V.M.); fmassari79@gmail.com (F.M.); 2 Medical Oncology Unit, Macerata General Hospital, 62100 Macerata, Italy; mattymo@alice.it

The advent of immunotherapy has revolutionized the treatment landscape of several hematological and solid tumors [1,2], and the number of patients eligible for immune-based cancer therapies continues to rise as these treatments are currently part of the first- or later-line standards for many malignancies [3,4,5]. Over the past decade, an impressive number of trials have been conducted on the role of immune checkpoint inhibitors, with the results of these studies paving the way towards the Immunotherapy Era in cancer care [6,7]. The first immune checkpoint inhibitor approved by the United States Food and Drug Administration (FDA) was ipilimumab, approved in 2011 for the treatment of advanced melanoma, following the results of the pivotal phase III trial conducted by Hodi and colleagues [8,9]. More than ten years after the publication of this study, there is a plethora of ongoing trials aiming to evaluate the efficacy and safety of immune checkpoint inhibitors, as a monotherapy or in combination with other anticancer agents [10,11]; thus, the number of cancer patients receiving immunotherapy is expected to further increase in the near future, especially considering that a wide range of combination therapies is being explored, with the aim of producing a synergistic effect [12,13].

For example, the treatment scenario of metastatic renal cell carcinoma (mRCC) has recently undergone a revolution following the results of landmark phase III clinical trials on immune checkpoint inhibitor–tyrosine kinase inhibitor combinations [14,15], such as pembrolizumab plus axitinib, nivolumab plus cabozantinib, pembrolizumab plus lenvatinib [16,17], as well as dual checkpoint bloackade with nivolumab plus ipilimumab in intermediate–poor-risk patients [18,19]. These treatments currently represent novel standards of treatment, as is recommended by the most up-to-date international guidelines [20,21].

However, several important issues have yet to be addressed in cancer immunotherapy. Among these, the efficacy of immune checkpoint inhibitors is considerably limited in some “immunologically cold” tumors (pancreatic adenocarcinoma, prostate cancer, biliary tract cancer, etc.), in which immunotherapy is still trying to find its therapeutic niche [22,23,24,25]. Moreover, and among the main challenges, the lack of predictive biomarkers that are able to guide therapeutic choices in several malignancies represents another important unmet need in this setting [26,27,28]. Several potentially useful predictors have been evaluated and are under assessment including, among others, PD-L1 expression, TMB, MSI, TILs and the gut microbiome [29,30,31].

This Special Issue aims to highlight key open questions and future perspectives in modern cancer immunotherapy, including novel immune checkpoint inhibitors and immune-based combinations, mechanisms of action (Figure 1), potential biomarkers predictive of response, experimental treatments, and other timely and emergent topics. International experts in this field will examine key approaches that are under active investigation in clinical and preclinical research, presenting critical analyses and integrating their own perspectives on promising drugs in clinical trials and the latest therapeutic strategies.

## Figures and Tables

**Figure 1:**
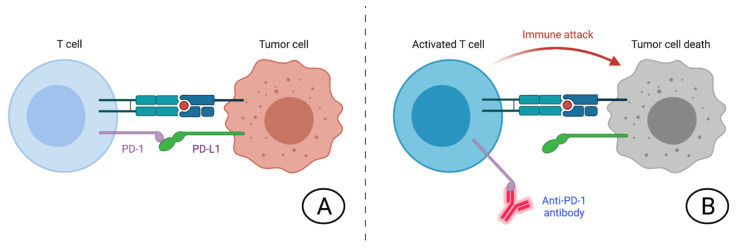
Schematic figure representing the mechanism of action of immune checkpoint inhibitors: (**A**) Immune checkpoint inhibits T-cell activation; (**B**) PD-1 inhibitors lead to T-cell activation.

## Data Availability

Not applicable.
